# Serum 25-hydroxyvitamin D is inversely associated with body mass index in cancer

**DOI:** 10.1186/1475-2891-10-51

**Published:** 2011-05-16

**Authors:** Pankaj G Vashi, Carolyn A Lammersfeld, Donald P Braun, Digant Gupta

**Affiliations:** 1 Cancer Treatment Centers of America® (CTCA) at Midwestern Regional Medical Center, 2520 Elisha Avenue, Zion, IL, 60099, USA

## Abstract

**Background:**

The association between vitamin D deficiency and obesity in healthy populations and different disease states remains unsettled with studies reporting conflicting findings. Moreover, current dietary recommendations for vitamin D do not take into account a person's body mass index (BMI). We investigated the relationship between serum 25-hydroxy-vitamin D [25(OH)D] and BMI in cancer.

**Methods:**

A consecutive case series of 738 cancer patients. Serum 25(OH)D was measured at presentation to the hospital. The cohort was divided into 4 BMI groups (underweight: <18.5, normal weight: 18.5-24.9, overweight: 25-29.9, and obese: >30.0 kg/m^2^). Mean 25(OH)D was compared across the 4 BMI groups using ANOVA. Linear regression was used to quantify the relationship between BMI and 25(OH)D.

**Results:**

303 were males and 435 females. Mean age at diagnosis was 55.6 years. The mean BMI was 27.9 kg/m^2 ^and mean serum 25(OH)D was 21.9 ng/ml. Most common cancers were lung (134), breast (131), colorectal (97), pancreas (86) and prostate (45). Obese patients had significantly lower serum 25(OH)D levels (17.9 ng/ml) as compared to normal weight (24.6 ng/ml) and overweight (22.8 ng/ml) patients; p < 0.001. After adjusting for age, every 1 kg/m^2 ^increase in BMI was significantly associated with 0.42 ng/ml decline in serum 25(OH)D levels.

**Conclusions:**

Obese cancer patients (BMI >= 30 kg/m^2^) had significantly lower levels of serum 25(OH)D as compared to non-obese patients (BMI <30 kg/m^2^). BMI should be taken into account when assessing a patient's vitamin D status and more aggressive vitamin D supplementation should be considered in obese cancer patients.

## Background

Serum 25-hydroxyvitamin D [25(OH)D] is the major circulating form of vitamin D and a standard indicator of vitamin D status [1,2]. Several studies have described an inverse relationship between serum 25(OH)D and cancer risk [3-5]. The relationship between regular vitamin D intake and reduced cancer incidence has also been reported [6]. Furthermore, higher plasma 25(OH)D levels are associated with improved survival in prostate [7], breast [8], lung [9], colorectal [10] and ovarian [11] cancers. A better vitamin D status at the time of diagnosis and treatment, adjusted for season of diagnosis, has been shown to improve survival [12,13].

Several factors are involved in the regulation of 25(OH)D including: age; gender [14]; race [15]; dietary intake [2]; season [16]; and sunlight exposure [17]. Recently, the relationship between obesity and vitamin D status has been investigated suggesting decreased bioavailability of 25(OH)D from cutaneous and dietary sources in association with obesity [18]. Additional studies have investigated the relationship between obesity and vitamin D levels. The results, however, are conflicting with studies reporting either no relationship [19], or an inversely proportional relationship [20-22] between body mass index (BMI) and serum 25(OH)D.

In studies of obese adolescents in the United States, vitamin D deficiency has been correlated with greater weight and elevated BMI [23,24]. In the healthy adolescent population the distribution of fat was found to be associated with vitamin D status with obese adolescents being found to have 25(OH)D deficiency [25]. Children with a body weight, BMI, bicipital skin fold thickness, waist measurement and waist/height ratio above the 50th percentile for each variable were found to be at a greater risk of having a low serum 25(OH)D concentration [26]. Similarly, body weight, BMI, and waist circumference of healthy women with higher vitamin D levels were smaller than those recorded for women with low vitamin D levels [27]. A statistically significant negative correlation between BMI and serum 25(OH)D in Caucasian and African Americans healthy adults has also been described [28].

An inverse relationship between obesity and serum levels of 25(OH)D has also been demonstrated in different disease states. In children with epilepsy, female sex and increased BMI were significant risk factors for low vitamin D levels [29]. In patients with Systemic Lupus Erythematosus, being overweight was found to be associated with vitamin D deficiency [30]. In another study, vitamin D deficiency was found to be more prevalent in morbidly obese patients presenting with the metabolic syndrome [31]. Finally, in women with polycystic ovarian disease low serum 25(OH)D concentrations were found as a result of obesity and insulin resistance [32].

The association between vitamin D and obesity assumes even greater importance in cancer, given the alleged role of both obesity and vitamin D in cancer risk and survival. Although it has been recommended that adiposity should be considered when assessing vitamin D requirements in obese patients [33], current dietary recommendations do not take into account a person's BMI and it remains unclear whether the dose of vitamin D required for repletion is related to the degree of obesity. And there are to date, no studies which indicate whether the presence of malignant disease compounds these issues. In the current study, we have addressed the first aspect of this question by investigating the relationship between serum 25(OH)D and BMI in a large and diverse population of cancer patients at a comprehensive cancer center.

## Methods

### Study Sample

This was a cross-sectional study performed on a consecutive case series of 738 cancer patients treated at Cancer Treatment Centers of America^® ^(CTCA) at Midwestern Regional Medical Center (MRMC) between January 2008 and June 2008. Only patients with a histologically confirmed diagnosis of cancer were included. The study examined the serum 25(OH)D levels of all new patients presenting to our institution as part of a routine nutritional status screening prior to initiation of anticancer therapy. The study did not restrict patients with respect to treatment history, tumor histology or stage. This study was approved by the Institutional Review Board at Midwestern Regional Medical Center (MRMC).

### Vitamin D Assessment

All patients underwent a baseline serum 25(OH)D assessment before undergoing any treatment at our hospital. Serum was collected, packed in coolpacks and sent to the Laboratory Corporation of America (Raleigh, NC) where a chemiluminescence immune assay (CLIA, DiaSorin Liasion assay) was used to measure 25(OH)D. Serum samples were incubated with antivitamin-D coated microparticles and isoluminol derivative-conjugated 25(OH)D before measurement of chemiluminescent signals. Analysis was completed within 48 hours of collection. The DiaSorin Liasion 25(OH)D assay has been clinically validated to be comparable in accuracy and precision to the radioimmunoassay (RIA). This method uses the same particles used in the DiaSorin RIA technique. Studies have found this to be a rapid, accurate, and precise tool for the measurement of serum 25(OH)D [34,35].

### BMI Assessment

At the subjects' first visit, measurement of height and weight were performed. The subjects wore light clothing and no shoes. BMI was calculated as weight (kg)/squared height (m^2^).

### Data Analysis and Statistical Methods

BMI was the independent variable and used as both continuous and categorical variables. Serum 25(OH)D was the dependent variable. Age at diagnosis and gender were used as covariates. Correlations between 25(OH)D and BMI as well as 25(OH)D and age were evaluated using Spearman's rho non-parametric correlation coefficient owing to their non-normal distributions. BMI was divided into 4 groups according to WHO criteria: <18.5 kg/m^2 ^(underweight), 18.5-24.9 kg/m^2 ^(normal weight), 25-29.9 kg/m^2 ^(overweight), and >30.0 kg/m^2 ^(obese). Mean 25(OH)D was compared across the 4 BMI groups using Analysis of Variance (ANOVA). Mean 25(OH)D levels were compared between males and females using 2 sample t-test. For the purpose of further exploring the relationship between BMI and serum 25(OH)D, we dichotomized 25(OH)D into 2 groups using a cut-off of 32 ng/ml. Patients with serum levels of <32 ng/ml were considered to have suboptimal serum 25(OH)D levels. Using chi square tests, we investigated the relationship between categorical BMI and dichotomous serum 25(OH)D. Univariate and multivariate linear regression analyses were used to quantify the relationship between BMI and 25(OH)D. Age, BMI and serum 25(OH)D were used as continuous variables for the purpose of regression analyses. All data were analyzed using SPSS 17.0 (SPSS Inc., Chicago, IL, USA). All analyses were two-tailed, and p values were considered significant when < 0.05.

## Results

### Baseline Characteristics

Table 1 describes the baseline characteristics of our patient cohort. The most common cancer types were lung, breast, colorectal, pancreas and prostate. The median age at presentation was 56 years (range 25 - 94 years). The mean BMI was 27.9 kg/m^2 ^(SD = 6.7 kg/m^2^). The mean serum 25(OH)D was 21.9 ng/ml (SD = 13.5 ng/ml).

**Table 1:** Baseline patient characteristics (N = 738)

Characteristic	Categories	Number	Percent (%)
Gender	Males	303	41.1
	Females	435	58.9

Age at Presentation (years)	Mean	55.6	
	Median	56.0	
	Range	25 - 94	

Cancer Site	Lung	134	18.2
	Breast	131	17.8
	Colorectal	97	13.1
	Pancreas	86	11.7
	Prostate	45	6.1
	Others	245	33.2

BMI Categories (kg/m^2^)	<18.5	23	3.1
	18.5 - 24.9	239	32.4
	25 - 29.9	249	33.7
	>= 30	227	30.8

### Univariate Analysis

The Spearman's rho correlation coefficient for BMI-25(OH)D association was -0.20 (p < 0.001) and for age-25(OH)D association was 0.1 (p = 0.009). Table 2 compares mean serum 25(OH)D levels across the 4 categories of BMI using ANOVA. Obese patients (>30.0 kg/m^2^) had statistically significantly lower serum 25(OH)D levels as compared to normal weight (18.5-24.9 kg/m^2^) and overweight (25-29.9 kg/m^2^) patients. Using 2 sample t-test, there was no significant difference found between mean 25(OH)D levels in males (21.8 ng.ml) and females (22 ng/ml); p = 0.83). Table 3 compares the prevalence of suboptimal serum vitamin D (<32 ng/ml) across the 4 categories of BMI. Obese patients had the highest prevalence of suboptimal vitamin D status. Table 4 provides the results of univariate linear regression analyses for age and BMI. Every 1 kg/m^2 ^increase in BMI was significantly associated with 0.43 ng/ml decline in serum 25(OH)D levels. Also, every one year increase in age at diagnosis was significantly associated with 0.11 ng/ml increase in serum 25(OH)D levels. It was decided to control for age in multivariate analysis.

**Table 2:** Mean serum 25(OH)D across 4 categories of BMI - univariate analysis (N = 738)

BMI (kg/m^2^)	Mean 25(OH)D (SD)	95% CI	ANOVA (P-Value)
1) < 18.5	22.4 (12.9)	16.8-28.1	10.5 (p < 0.001)
	
2) 18.5 to 24.9	24.6 (15.9)	22.6-26.7	
	
3) 25 to 29.99	22.8 (12.6)	21.2-24.4	
	
4) >= 30	17.9 (10.6)	16.6-19.4	

Post-hoc tests revealed significant differences between 2 and 4 &3 and 4 BMI categories

**Table 3:** Relationship between categorical BMI and dichotomous serum 25(OH)D (N = 738)

BMI (kg/m^2^)	Suboptimal 25(OH)D <32 ng/ml	Optimal 25(OH)D >= 32 ng/ml	Chi Square (P-Value)
< 18.5	18 (78.3)	5 (21.7)	
	
18.5 to 24.9	177 (74.1)	62 (25.9)	21.2 (p < 0.001)
	
25 to 29.99	206 (82.7)	43 (17.3)	
	
>= 30	205 (90.3)	22 (9.7)	

The numbers in parenthesis are row percentages

**Table 4:** Univariate linear regression analysis with serum 25(OH)D as the dependent variable (N = 738)

Variable	B Coefficient (Standard Error)	95% CI	P - Value
BMI	-0.43 (0.07)	-0.57 to -0.29	<0.001

Age	0.11 (0.05)	0.009 to 0.20	0.03

Gender (male as reference)	0.22 (1.0)	-1.8 to 2.2	0.83

### Multivariate Analysis

Table 5 displays the results of multivariate regression analysis for BMI-25(OH)D association after controlling for the effects of age at diagnosis. Every 1 kg/m^2 ^increase in BMI was significantly associated with 0.42 ng/ml decline in serum 25(OH)D levels after adjusting for age. Age was no longer significant in the final model.

**Table 5:** Multivariate linear regression analysis with serum 25(OH)D as the dependent variable (N = 738)

Variable	B Coefficient (Standard Error)	95% CI	P - Value
BMI	-0.42 (0.07)	-0.56 to -0.28	<0.001

Age	0.08 (0.05)	-0.009 to 0.18	0.08

The BMI-25(OH)D association was further explored in each of the top 5 cancer sites including lung, breast, colorectal, pancreas and prostate. A statistically significant relationship was observed in breast and colorectal cancers only. After adjusting for age, every 1 kg/m^2 ^increase in BMI was significantly associated with 0.51 ng/ml (p = 0.02) and 0.46 ng/ml (p = 0.006) decline in serum 25(OH)D levels in breast and colorectal cancers respectively.

## Discussion

Characterization of serum vitamin D levels in cancer patients is of considerable importance given the role of vitamin D in influencing cancer risk and survival. An important question that arises when recommending vitamin D supplementation in deficient cancer patients is whether the dose should be adjusted according to a patient's BMI. While there is some evidence in the literature documenting an inverse relationship between BMI and serum 25(OH)D in healthy and diseased populations, this relationship in cancer has never been reported. Thus, the current study was undertaken to investigate this relationship. The results demonstrate that serum 25(OH)D levels are significantly lower in obese cancer patients as compared to normal and overweight patients, and that every unit increase in BMI is associated with a corresponding decrease in serum 25(OH)D that is statistically significant.

Multiple mechanisms have been proposed to explain the association of obesity with hypovitaminosis D, including lack of sunlight exposure from physical inactivity [36] and sequestration of vitamin D in subcutaneous fat depots [33]. Wortsman et al. reported that the capacity of the skin to produce vitamin D is not altered in obesity. However, the increase in serum vitamin D after sun exposure was 57% less in obese compared with non-obese subjects suggesting a decreased bioavailability of vitamin D because of its deposition in body fat compartments, given that vitamin D is fat soluble [18]. Young KA et al. observed that higher BMI and subcutaneous adipose tissue were associated with lower 1,25-dihydroxyvitamin D [1,25[OH]2D], after controlling for 25[OH]D levels, suggesting a more complex relationship between vitamin D and adiposity than simply attributing this association to vitamin D bioavailability [37]. Another hypothesis suggests a negative feedback inhibition of 25(OH)D synthesis in liver by the increased level of 1,25(OH)2D [32,38]. Bell NH et al. reported that alteration of the vitamin D endocrine system in obese subjects is characterized by secondary hyperparathyroidism which is associated with enhanced renal tubular reabsorption of calcium and increased circulating 1,25(OH)2D which causes a feedback inhibition of 25(OH)D synthesis [39]. However, some recent studies have reported an inverse correlation between BMI and serum 1,25(OH)2D, casting a doubt on the hypothesis of negative feedback inhibition [21,28].

There are obvious clinical implications of this work such as the need to monitor the vitamin D intake and serum 25(OH)D levels in patients with cancer and supplementation of vitamin D in those who are found to have suboptimal levels. In our recently published study in a sample of 799 cancer patients, we found that patients with suboptimal vitamin D levels at baseline, when supplemented with 8000 IU of Vitamin D3 (four 2000 IU D3 capsules) daily as part of their nutritional care plan, showed significant improvement in their serum vitamin D levels after a mean follow-up of 14.7 weeks [40]. A study conducted by Lee P et al. in 95 out-patients found that increments of vitamin D level were less in obese individuals, implying that a larger dose of vitamin D supplementation is required for repletion compared with normal-weight individuals [33]. Similarly, Jorde et al. investigated the serum 25(OH)D response to vitamin D supplementation in relation to BMI and concluded that when giving vitamin D supplements to the very obese, the dose has to be higher than in the lean subjects if the same serum 25(OH)D levels are to be achieved [41]. A study conducted in postmenopausal women by Moschonis G et al. suggested a need for higher daily vitamin D intake in obese than in thinner individuals to compensate for the lower bioavailability of endogenously produced cholecalciferol [42]. Blum M et al. found that change in 25(OH)D levels in response to vitamin D supplementation was inversely associated with BMI in healthy men and women 65 years of age and older. As a result, they recommend that that body size should be taken into account when estimating the amount of vitamin D intake needed to raise 25(OH)D to the desired level [43]. The most recent report by the Institute of Medicine recommends a dietary reference intake of 600 IU for vitamin D [44]. The results of our study, coupled with the findings reported by other researchers as described above, provide preliminary evidence to suggest that obese people might require a higher dose of vitamin D to achieve optimal serum levels.

Interestingly, we found a weak but significantly positive association between age and 25(OH)D levels. This is unexpected since the efficiency of the photosynthesis of vitamin D in human skin decreases with age [45]. In addition, older people may receive less sunlight exposure because of frailty and reduced exercise levels or for cultural and behavioral reasons [46]. The literature in this regard is inconsistent with reports being presented purporting to demonstrate a positive relationship [45,47], no relationship [48], and an inverse relationship [49,50] between age and 25(OH)D level. Although it is reasonable to postulate that differences in life style and dietary habits will explain these inconsistencies, the reason for these divergent results remains unclear and warrants rigorously controlled studies.

Our study has some limitations. This study, because of its retrospective nature, relies on data not primarily meant for research. As a result, we could not adjust for several potential confounding factors that could have influenced serum 25(OH)D levels. For example, we did not adjust for season of blood draw in our analyses. However, our analysis was limited to patients tested in late winter, spring and early summer, so a major difference in sun exposure seems unlikely to account for the differences in the serum vitamin D levels of our patient population. We included only one serum sample from each subject. Intervention and interpretation of serum 25(OH)D levels might require multiple measurements over a period of time. Moreover, we did not have information on intake of vitamin D or data regarding their typical sun exposure which could have shed further light on the subjects' vitamin D status.

Other variables such as race, physical activity and cancer treatment history are all thought to affect 25(OH)D status but were not controlled for in the analyses. Because anthropometric indicators of adiposity such as BMI are traditionally weaker than direct measures of adiposity, it is likely that we are underestimating the association between vitamin D and overall adiposity by using BMI as a measure of obesity [37]. However, BMI is non-invasive, low cost and east to perform. Despite these limitations, our study provides evidence of an inverse relationship between serum vitamin D and BMI in cancer patients - a novel finding in the oncology patient population. Strengths of the present investigation are its relatively large sample size and the fact that the vitamin D analyses were all performed using the same assay.

The results of this study suggest several areas for future research. Paramount among these is the immediate need to determine the response to vitamin D supplementation in cohorts of patients segregated according to BMI or other measures of obesity. Such studies are well within the capacity of clinical investigators and are critical to establishing optimal vitamin D dose and schedules for adequate repletion. The results of such studies should support further investigation of the consequences of vitamin D repletion on clinical outcomes in cancer patients pertinent to either specific functions (e.g. treatment response, wound healing) or more general outcomes such as prognosis and QoL. Ultimately, such studies should determine whether restoration and maintenance of adequate vitamin D levels can influence tumor control and survival. Although the current study was conducted in a large heterogeneous cancer population, the same approaches would be appropriate for elucidating the importance of vitamin D levels in homogeneous patient populations segregated according to tumor type and treatment selection.

## Conclusions

We found that obese cancer patients (BMI >= 30 kg/m^2^) had significantly lower levels of serum 25(OH)D as compared to non-obese patients (BMI <30 kg/m^2^). BMI should be taken into account when assessing a patient's vitamin D status and more aggressive vitamin D supplementation should be considered in obese cancer patients.

## Competing interests

The authors declare that they have no competing interests.

## Authors' contributions

PGV participated in concept, design, data collection, data analysis, data interpretation and writing. CAL participated in data collection, data interpretation and writing. DPB participated in writing, data interpretation and general oversight of the study. DG participated in data collection, data analysis, data interpretation and writing. All authors read and approved the final manuscript.
